# Post-weaning diarrhea in pigs weaned without medicinal zinc: risk factors, pathogen dynamics, and association to growth rate

**DOI:** 10.1186/s40813-021-00232-z

**Published:** 2021-10-09

**Authors:** Esben Østergaard Eriksen, Egle Kudirkiene, Anja Ejlersgård Christensen, Marianne Viuf Agerlin, Nicolai Rosager Weber, Ane Nødtvedt, Jens Peter Nielsen, Katrine Top Hartmann, Lotte Skade, Lars Erik Larsen, Karen Pankoke, John Elmerdahl Olsen, Henrik Elvang Jensen, Ken Steen Pedersen

**Affiliations:** 1 Department of Veterinary and Animal Sciences, University of Copenhagen, Copenhagen, Denmark; 2 Ø-Vet A/S, Næstved, Denmark; 3 SEGES Danish Pig Research Centre, Copenhagen, Denmark; 4 Department of Production Animal Clinical Sciences, Norwegian University of Life Sciences, Ås, Norway

**Keywords:** Post-weaning diarrhea, Weaning, Pig, Rotavirus, *E. coli*, Enterotoxigenic, Weight gain

## Abstract

**Background:**

Porcine post-weaning diarrhea (PWD) has reemerged as an important topic in pig production, as common control strategies based on prophylactic use of antimicrobials and zinc oxide have been deemed unsustainable. The objectives of this study were to estimate the cumulative incidence of porcine post-weaning diarrhea with different etiologies in production systems weaning without zinc oxide and prophylactic antimicrobials, to assess risk factors for post-weaning diarrhea, and to estimate the impact of post-weaning diarrhea on growth rate. A cohort study was conducted at two commercial indoor producers weaning without medicinal zinc oxide and prophylactic antimicrobials.

**Results:**

Piglets were included at birth (n = 300) and 272 survived until weaning. After insertion to the nursery units, the piglets were clinically examined every day for 14 days, and rectal swabs were collected and analyzed for enterotoxigenic *Escherichia coli* (ETEC) and rotavirus A. The cumulative incidences of PWD the first 14 days after insertion to the nursery units were 41.8% (CI 33.6, 50.4) and 51.1% (CI 42.3, 60.0) at the two producers, respectively. We found a low incidence of cases associated to ETEC, and detected a substantial proportion of cases associated to rotavirus. We observed a biphasic pattern in the assumed etiology with rotavirus occurring first, and then a shift towards cases associated to ETEC/non-ETEC hemolytic *E. coli.* Being offspring of older sows was a protective factor for the development of PWD (Hazard ratio = 0.88 [CI 0.78, 0.99] per unit increase in parity of the dam). Low birth weight reduced the post-weaning growth rate (− 5.2 g/day [CI − 7.5, − 2.9] per 100 g decrease in birthweight) and increased the hazard of developing PWD (Hazard ratio for birthweight below 1100 g: 2.30 [CI 1.41–3.74]). The combined effect of having diarrhea for 2 days or more and receiving antimicrobial treatment was associated with an increased average daily weight gain.

**Conclusions:**

This study suggests novel insights regarding pathogen dynamics and risk factors for PWD in productions not using prophylactic antimicrobials and medicinal zinc. The findings may have important implications for both antimicrobial usage and prevention strategies.

**Supplementary Information:**

The online version contains supplementary material available at 10.1186/s40813-021-00232-z.

## Introduction

Weaning of pigs is a delicate process where suckling is terminated and solid food replaces the milk-based diet. If humans do not intervene, piglets of the domesticated swine will gradually undergo this transition completing it when approximately 15–22 weeks old [1]. In contrast, the weaning takes place as an abrupt event in the intensive indoor pig production. Under common Danish and European intensive production schemes, piglets are removed from the sow and weaned to special nursery units at 3–5 weeks of age. At this age the colostrogenic immunity is declining and the lactogenic immunity is lost from the discontinued suckling [2, 3]. Furthermore, the piglets have an immature gut with a sub-optimal microbial community, an undeveloped mucosal-immunity and an impaired intestinal epithelial barrier function [4–7], and they are not adapted to solid feed. In addition, the gut health might be further compromised by the stressful events during weaning [8, 9], and the new environment where surface contamination and pen mates might introduce the piglets to new pathogens. This leaves the piglets in a very vulnerable position.

Post-weaning diarrhea (PWD) is a multifactorial condition occurring the first 14 days after weaning where the clinical sign diarrhea (i.e. defecation with increased rate, volume and water content) is resulting from the aforementioned factors commonly enhanced by an infection with specific pathogens. The most important pathogen in this regard is reported to be enterotoxigenic *Escherichia coli* (ETEC) [10]. However, PWD with no detection of ETEC is common [11], and it has been suggested that an intestinal dysbiosis in itself might cause intestinal inflammation and diarrhea after weaning in pigs [4]. Rotavirus is another commonly occurring [12] and contributing pathogen [13, 14]. Porcine enteric coronaviruses are also important pathogens [15], but Denmark is declared free from these [16].

Traditionally, pig producers have succeeded with weaning at an early age with limited signs of gastrointestinal disease shortly after weaning. This has to a large extent been possible by the provision of oral antimicrobials [17] and high doses of in-feed medicinal zinc oxide [18–20]. However, the use of antimicrobials poses a risk to both human and animal health by selecting for antimicrobial resistance [21, 22]. Likewise, medicinal zinc oxide co-selects for antimicrobial resistance, and the use possess an environmental hazard, as most of the zinc is excreted with the feces and through manuring it accumulates in soil [23]. Accordingly, medicinal zinc oxide has been prohibited in the European Union effectively coming into force no later than June 2022 [24]. Antimicrobial growth promoters have been banned in the European Union since 2006 [25] and lately other parts of the world [26], and prophylactic use of antimicrobials is also prohibited in many European countries including Denmark [27]. Therapeutic and metaphylatic use of antimicrobials remains an option [27]. However, there is a strong demand for reduction of the use by the public, the consumers, and the authorities globally [28–30]. Consequently, the pig industry must adopt novel strategies for the control of PWD.

The porcine intestinal disease complex occurring in older nursery pigs [31] sometimes have a low-pathogenic nature [32], in which case antimicrobial batch medications cannot be justified [32–34]. Analogously, pigs, which are not given medicinal zinc oxide, might experience PWD where antimicrobial batch medications cannot be justified. Previous studies (e.g. [11]) have indicated that ETEC is not necessarily present in all PWD outbreaks, and this should be further investigated. As a key for prevention, risk factors for PWD in such pigs should also be determined. Furthermore, impaired gut health can be associated with reduced productivity, which is undesirable from the perspective of resource use, climate gas emissions, and production economy [35–37].

Therefore, the present study investigated piglets raised at commercial Danish intensive indoor producers weaning without medicinal zinc and prophylactic antimicrobials. The objectives were to estimate the cumulative incidence of pigs which develop clinical signs of diarrhea associated with a bacterial intestinal infection during the first 14 days after insertion to the nursery unit, to assess early-life risk factors for post-weaning diarrhea, and to investigate the association between post-weaning diarrhea and growth rate.

## Results

### Description of the study population

This cohort study encompassed piglets from 30 L located in two commercial pig producers in Denmark during September and October 2019. The litters consisted of 90% live born piglets, the median litter size was 23 (range: 14–28), and the parity of the sows ranged from one to seven. Details of these parameters are summarized in Additional file 1: Additional table A. In Table 1, we have summarized descriptive parameters of the cohort followed at the two producers.

**Table 1:** Summary of the piglets included in a cohort study of post-weaning diarrhea

	Producer A	Producer B
Sex at birth		
Female	64/150 (43.7%)	72/150 (48.0%)
Male	86/150 (57.3%)	78/150 (52.0%)
Sex at insertion^a^		
Female	61/141 (43.2%)	63/131 (48.1%)
Male	80/141 (56.7%)	68/131 (51.9%)
Pre-weaning mortality rate^b^	9/150 (6.0%)	19^c^/150 (12.7%)
Moved to foster sow	113/149^e^ (75.8%)	74/148^f^ (50%)
Weaned in the farrowing unit^d^	61/140^e^ (43.6%)	51/131 (38.9%)
Mean birth weight (g)	1345 (SD: 316)	1344 (SD: 302)
	Range: 540–2080	Range: 690–2070
	n = 150	n = 150
The mean body weights at insertion to the nursery units (g)	6054 (SD: 1670)	6549 (SD: 1484)
	Range: 2100–11,500	Range: 2900–11,000
	n = 140^e^	n = 131
Average daily weight gain the first 14 days after insertion to the nursery units (g)	155 (SD: 59 g)	168 (SD: 55)
	Range: 14–300	Range: 36–321
	n = 133	n = 125^e^

^a^Sex at insertion was recoded to be in accordance with the sex registered at birth whenever these registrations did not agree (n = 2)

^b^Dead/euthanized by herd personnel

^c^Including one pig lost from follow up that was assumed to be dead

^d^Before the common weaning day

^e^Data from one piglet was missing

^f^Data from two piglets were missing

One piglet was missing at insertion to nursery unit B, and therefore assumed dead in the suckling period. Apart from this, all 300 piglets were followed throughout the study period. As seen in Table 1, sex was distributed approximately evenly at birth at both producers. At insertion to the nursery unit, the males had been castrated.

Among all piglets (n = 300), three had missing registration of which sow/farrowing pen they were housed in at the time of death (n = 2) or weaning (n = 1). As seen in Table 1, a majority (n = 187/297) was found at a foster sow instead of the dam.

Both producers practiced routine IM injections with antimicrobials during the first day of life to prevent neonatal infections (Producer A: dihydrostreptomycin 31.25 mg and benzylpenicillin procaine 25,000 UI per 1 kg bodyweight [Streptocillin® vet., Boehringer Ingelheim]. Producer B: Amoxicillin trihydrate 37.5 mg/pig, [Clamoxyl® Prolongatum Vet., Zoetis]). Apart from this, 24 piglets received an antimicrobial treatment before weaning at producer A; six of these were against diarrhea. Seven pigs were subjected to an antimicrobial treatment before weaning at producer B.

**Fig. 1:**
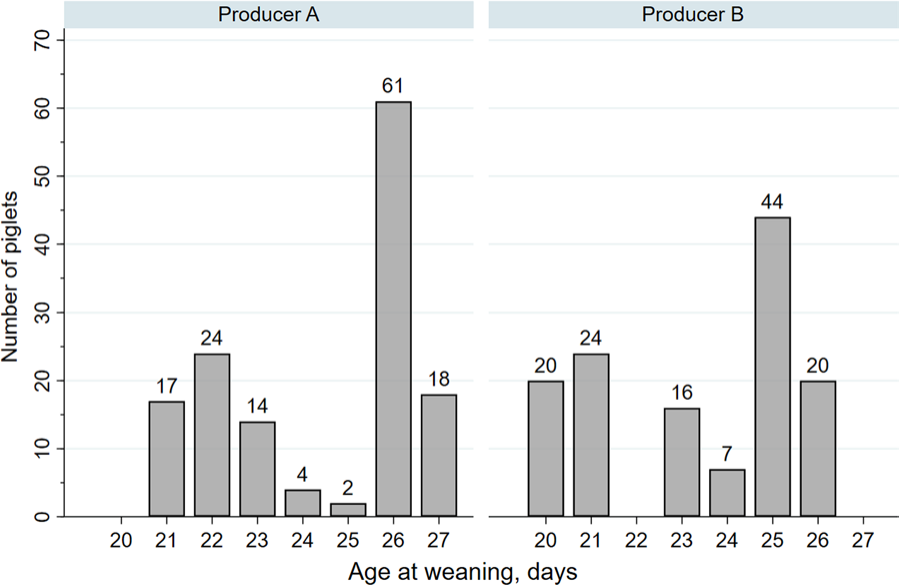
Age at weaning in two cohorts of piglets followed at two Danish indoor producers

We aimed at not interfering with the producers’ usual management practices. Hence, herd personnel made decision about weaning piglets if a sow were to be used as a foster sow. Consequently, a substantial proportion was weaned earlier than common weaning day at both producers (Table 1). The first piglets were weaned at study day 21 and 20 at producer A and B respectively, and the distribution of weaning age is displayed in Fig. 1 (note that weaning before 21 days of age is prohibited unless due to health or welfare issues, see ethical statement). The age at weaning was most commonly 26 days and 25 days at the two producers, respectively.

#### Mortality rates and causes of death

The pre-weaning mortality rates are presented in Table 2. The most common causes of death were non-infectious i.e. crushing by the sow (n = 10) and starvation (n = 4). Starvation was only recorded at producer B. Infectious causes of death included polyserositis (n = 1), enteritis, enterocolitis (n = 2), polyarthritis (n = 2) and osteomyelitis (n = 1). In six of the pigs a cause of death could not be stated due to lack of pathological changes or because the autolytic changes made the pathological evaluation impossible. A complete table of causes of death and associated pathological manifestations is included in Additional file 1: Additional table 2. No pigs died after weaning, but for animal welfare reasons, four pigs in poor general condition were euthanized by the investigators during the 14 days the pigs were followed in the nursery (producer A n = 3, producer B n = 1).

**Table 2:** Toxin and fimbriae types of hemolytic *E. coli* from weaned Danish piglets

Genotype	Producer A		Producer B	
	All isolates^a^	New (first or second) diarrhea cases	All isolates^a^	New (first or second) diarrhea cases
F18 + STa + STb	144	21	4	1
F18 + STb + LT	0		16	7
F4 + STb + LT	0		3	2
F18	0		118	24
STa + STb	0		1	1
STb + LT	0		1	1
STb	4	1	5	2
No fimbria or toxins	19	3	49	4
Total	167		193	
Missing values^b^	17	2	19	11

Isolates were cultured from rectal swabs collected within the first 14 days after insertion to the nursery unit, and tested for F18 and F4, and STa, STb and LT

^a^All isolates including both systematically sampled pigs and samples from diarrhea cases

^b^PCR results from 34 samples, where hemolytic *E. coli* was deemed dominant were missing. Of these, 13 were from new cases of diarrhea

### Occurrence of diarrhea

**Fig. 2:**
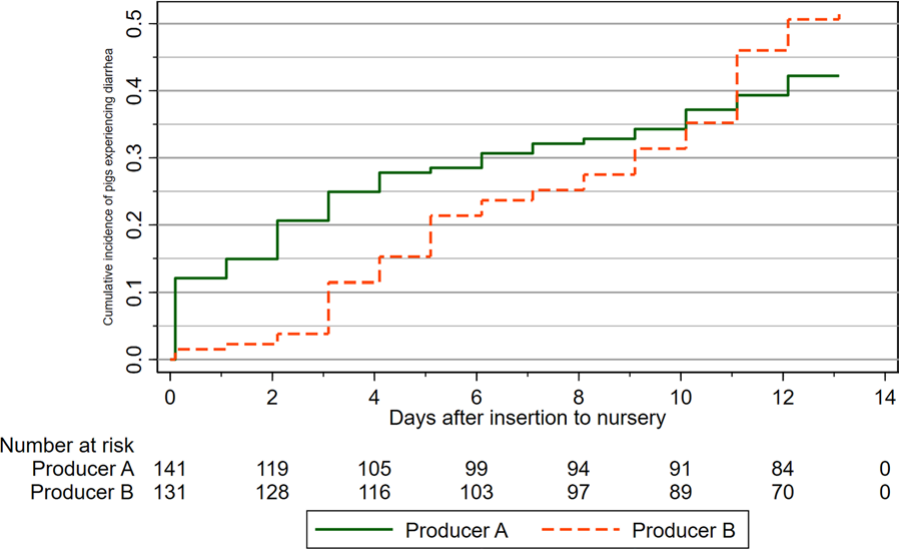
The cumulative incidence of diarrhea after insertion into the nursery unit. Pigs were clinically examined every day at two Danish indoor producers (A, B), and cumulative incidences were estimated using a Kaplan–Meier failure function. All pigs (n = 141, Producer A. n = 131, Producer B) were assumed to be non-diarrheic at the time of insertion (time = 0), and the first clinical exam was performed at time = 0.1

**Fig. 3:**
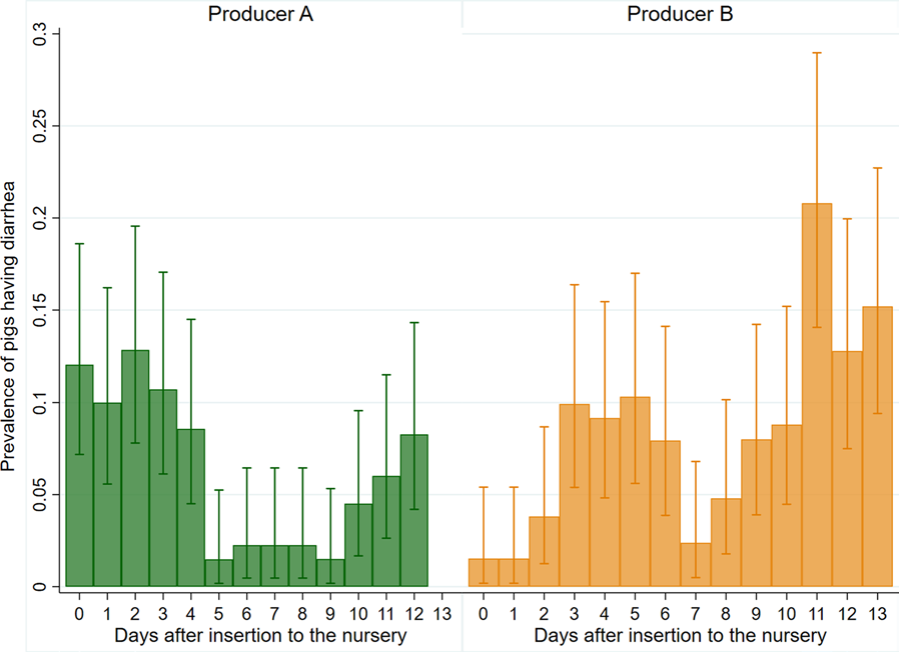
Prevalence of diarrhea during the first 14 days after insertion in the nursery unit. Figure legend: Prevalence proportion with 95% confidence intervals (Clopper-Pearson) of pigs having diarrhea during the first 14 days after insertion in the nursery unit in two Danish indoor productions (**A**, **B**). n = 131 and n = 142 at day 0, and n = 133 and n = 125 at day 13 in the two nurseries A and B, respectively. The prevalence was 0 at day 13 in nursery A

In nursery A, 59 piglets experienced at least one case of diarrhea, and 67 pigs did so in nursery B. The corresponding cumulative incidence of diarrhea during the first 14 days after insertion in the nursery was 41.8% (CI 33.6, 50.4) in nursery A (n = 141) and 51.1% (CI 42.3, 60.0) in nursery B (n = 131). The incidence rate of first cases of diarrhea was 4.55 (CI 3.52, 5.87) diarrhea cases/100 piglet days in nursery A, and 5.06 (CI 3.98, 6.43) diarrhea cases/100 piglet days in nursery B. Kaplan–Meier failure estimates for first cases of diarrhea is presented for each producer in Fig. 2. At producer A, eight pigs experienced a second case of diarrhea, while 16 did so at producer B. The prevalence proportions of pigs suffering from diarrhea during the 14 days are displayed in Fig. 3.

### Microbiology

Samples were collected in cases of diarrhea for all pigs in the cohort, and additionally half of the piglets were systematically sampled for bacteriology every second day. Bacterial isolation descriptions were missing for 11 systematic sampling points in nursery A and two in nursery B, and isolation descriptions were missing for two and five first cases of diarrhea samplings in nursery A and B respectively. PCR was used to determine toxin and fimbriae profiles of *E. coli* isolates. Results from the PCR lacked for 36 isolates that were hemolytic *E. coli* and which fulfilled the criteria for abundant growth. Of these missing values, 13 originated from first (n = 11) or second (n = 2) incidences of diarrhea, and 23 from the systematic sampling. Among the 360 hemolytic *E. coli* with available PCR results, F18 + STa + STb (n = 144) was the most common genotype in nursery A, and F18 without the investegated toxins (n = 118) was the most common genotype in nursery B (Table 2).

**Fig. 4:**
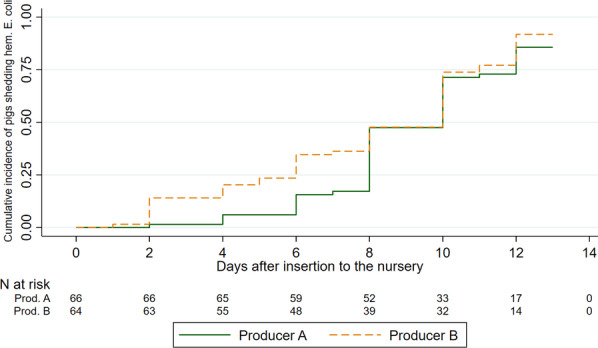
The cumulative incidence of hemolytic *E. coli* shedding after insertion into the nursery unit. Figure legend: The cumulative incidence (Kaplan–Meier failure estimates) of fecal shedding of hemolytic *E. coli* as a function of time since insertion in the nursery unit (days) at two Danish indoor productions (A, B). The pigs were systematically sampled every second day. Failure was defined as: Rectal swabs cultured on blood agar displayed abundant growth of hemolytic *E. coli*-like colonies

**Fig. 5:**
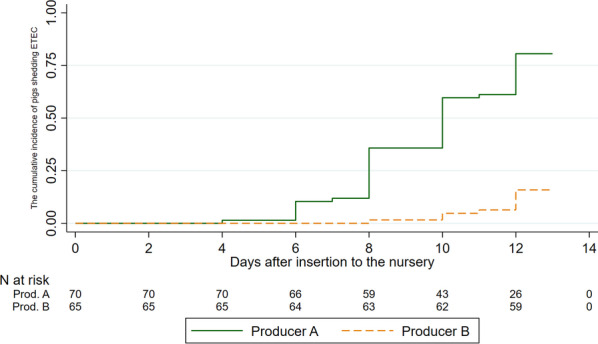
The cumulative incidence of enterotoxigenic *E. coli* shedding after insertion into the nursery unit. The cumulative incidence (Kaplan–Meier failure estimates) of fecal shedding of enterotoxigenic *E. coli* (ETEC) as a function of time since insertion in the nursery unit (days) at two Danish indoor productions (A, B). The pigs were systematically sampled every second day. Failure was defined as: Rectal swabs cultured on blood agar displayed overgrowth of hemolytic colonies confirmed to be ETEC by PCR

The systematic sampling offered a basis for mapping of *E. coli* shedding over time. Figure 4 presents the cumulative incidence of pigs shedding hemolytic *E. coli* to an extent where the defined quantitative criteria for “abundant growth” was fulfilled. It is seen that almost all pigs excreted *E. coli* above this level in at least one occasion during the first 13 days after insertion. Likewise, the shedding of ETEC was also mapped (Fig. 5). To be considered a failure event in this regard, the defined quantitative criteria were still applied, and ETEC was defined as having at least one fimbria (F4 or F18) gene and at least one toxin (STa, STb or LT) gene. There was no substantial shedding of ETEC the first 6 days after insertion. Interestingly, the frequent shedding of hemolytic *E. coli* in nursery B (Fig. 4) did not reflect a frequent shedding of ETEC (Fig. 5 and Table 2).

The effect of the *E. coli* virulence factors was explored by comparing the incidence rates of diarrhea cases between pigs shedding *E. coli* strains with different profiles of hemolytic ability, fimbriae and toxins. A Poisson regression model was based on 33 cases occurring during 941 pig days with 455 bacteriological recordings, and the results are displayed in Table 3. Nine additional cases had missing values for the virulence profile of *E. coli* on the case-day, and these were omitted from the analysis. The modelling indicated that new cases of diarrhea occurred at a lower rate in pigs shedding hemolytic *E. coli* that were fimbria positive but lacked toxins, compared to pigs shedding enterotoxigenic *E. coli* carrying both fimbria and toxin genes (IRR = 0.25 [CI 0.09–0.68]) (Table 3). Diarrhea occurred at a greater rate in pigs shedding hemolytic *E. coli* carrying fimbria but no toxins compared to pigs not shedding hemolytic *E. coli*.

**Table 3:** Incidence rate ratios of diarrhea cases in pigs shedding *E. coli* with different virulence factors

	Cases/pig days	Incidence rate ratio	95% Confidence Interval		Std. Err	*p* value
Baseline incidence rate (constant)		0.06	0.03	0.11	0.02	5.5e^−22^
Producer						
Producer A	12/415	1.00 (base)				
Producer B	18/385	3.78	1.85	12.83	2.41	0.001
Virulence factors						
H+, F+T+	17/209	1.00 (base)				
H+, F−, T+	1/5	1.87	0.25	14.24	1.94	0.544
H+, F+, T−	11/150	0.25	0.09	0.68	0.13	0.007
H+, F−, T−	2/48	0.10	0.033	0.01	0.11	0.033
H−	0/361	NA^a^				

Incidence rate ratios were estimated with a Poisson regression model. *E. coli* was cultured from rectal swabs collected from a cohort of piglets raised at two commercial Danish indoor producers. Data was included from the seventh to the 14th day after insertion to the nursery units

H+/H−, hemolytic *E. coli* positive/negative; F+/F−, fimbria positive/negative; T+/T−, toxin positive/negative

^a^Incidence rate was 0

### Diarrhea cases sorted on assumed etiology

**Fig. 6:**
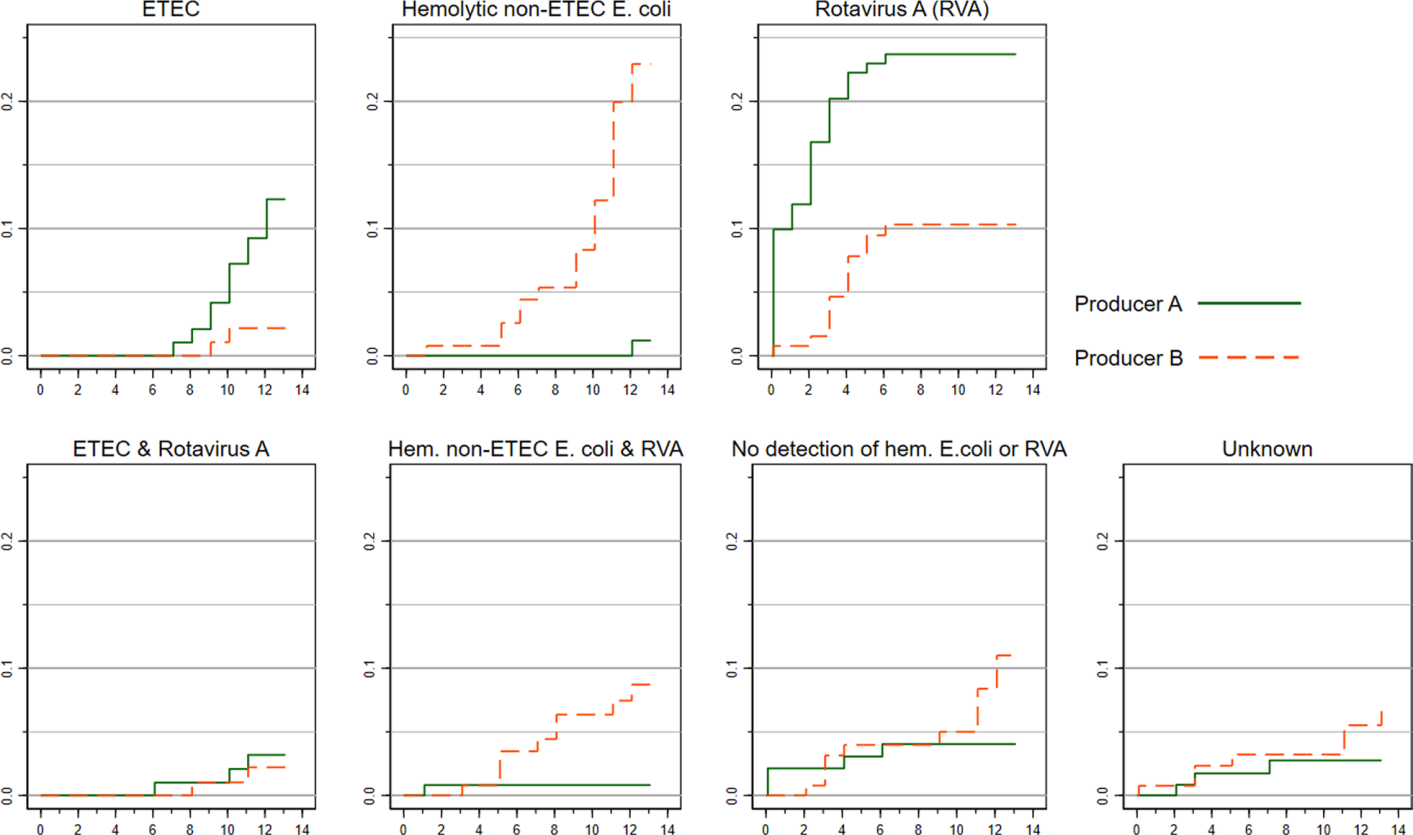
The cumulative incidence of diarrhea after insertion in the nursery unit divided on etiologies. The cumulative incidence (Kaplan–Meier failure estimates, y-axes) of diarrhea divided on etiologies (7 different plots) as a function of time since insertion to the nursery unit (days) (x-axes) at two Danish indoor productions (A, B). In these estimates, all pigs were assumed to be healthy at insertion (t = 0), and thus prevalent diarrhea cases at the first clinical exam (t = 0.1) were included as a failure (as opposed to left truncating them). N at risk at t0 = 141 (producer A) and 131 (Producer B)

The diarrhea cases were sorted on assumed etiologies according to the definitions described in the Methods paragraph. For this purpose, imputations were made for 11 missing values of ETEC and four missing value for rotavirus A. Reasonable imputations could not be made for 10 pigs with missing values, and thus they received the diagnosis *unknown etiology*. Table 4 summarizes the number of diarrhea cases with different assumed etiologies and their mean time-to-occurrence in days. In nursery A, rotavirus A was the dominating etiology being detected in 38 cases (64.4%). In Nursery B, an abundant growth of hemolytic non-ETEC *E. coli* was commonly seen (n = 32, 47.7%) in association with the first diarrhea case. The incidences and dynamics of different etiologies are seen from the plots of Kaplan–Meier failure estimates divided on the different etiologies in Fig. 6. Clearly, the first week was dominated by rotavirus-associated cases (median time-to-occurrence = 3 days, Table 4). In the second week, the incidence of diarrhea associated to ETEC (nursery A) and hemolytic non-ETEC *E. coli* (nursery B) accelerated. Meanwhile, rotavirus was rarely detected in high quantities (CT < 33) in the diarrheic pigs and never as the sole pathogen.

**Table 4:** Time-to-event and producer-wise distribution of first cases of post-weaning diarrhea with different assumed etiologies

Assumed etiology at first cases of diarrhea	Time to diarrhea event (days)^a^			Producer A		Producer B	
	25th pctl	Median	75th pctl	n	%	n	%
ETEC	9	10	11.25	12	20.3	2	3
Hemolytic non-ETEC *E. coli*	7.5	10	11	1	1.7	23	34.3
Rotavirus A	2	3	4	34	57.6	13	19.4
ETEC and rotavirus A	7	10	11	3	5.1	2	3
Hemolytic non-ETEC *E. coli* and rotavirus A	4.5	6	8.75	1	1.7	9	13.4
No detection of hemolytic *E. coli*, ETEC or rotavirus A^b^	3	6	11	5	8.5	11	16.4
Unknown etiology (missing data)	3	5	11	3	5.1	7	10.5

Summary of post-weaning diarrhea cases with different assumed infectious etiologies occurring in a cohort of piglets followed for 14 days after insertion into two Danish indoor nursery units. Data is presented as the median, 25th and 75th percentiles of the time to first diarrhea event, and as the number and percentage of first events of diarrhea at the two different producers (producer A and B)

^a^The prevalent cases at the day of insertion were not included in the calculations

^b^Rotavirus, hemolytic *E. coli* & ETEC were detected in some of these pigs, but below the defined threshold (CT > 33) or the semi-quantitative criteria for dominance

### Time to cure

The time to cure after the first diarrhea event is summarized in Table 5. The cure-rate was similar across both producers and assumed etiology. Just over half of the piglets were already cured 1 day after the treatment initiation, and almost all pigs were cured within 3 days.

**Table 5:** Proportion (standard error) of pigs cured from post-weaning diarrhea after a given of number days

	1 day	2 days	3 days	4–8 days
By producer				
Producer A (n = 38)	0.61 (0.079)	0.91 (0.034)	0.997 (0.003)	1
Producer B (n = 59)	0.53 (0.065)	0.85 (0.036)	0.98 (0.010)	> 0.998 (< 0.002)
By etiology				
ETEC (n = 14)	0.5 (0.13)	0.95 (0.045)	0.995 (0.007)	> 0.999 (0.002)
Hemolytic non-ETEC *E. coli* (n = 24)	0.5 (0.10)	0.82 (0.064)	0.96 (0.025)	> 0.993 (0.059)
Rotavirus A (n = 25)	0.48 (0.10)	0.79 (0.65)	0.98 (0.012)	1

The cure-ratios were estimated using a Kaplan–Meier failure function. The estimates were stratified by producer (A or B) and by three different assumed infectious etiologies

### Risk factors for post-weaning diarrhea

**Fig. 7:**
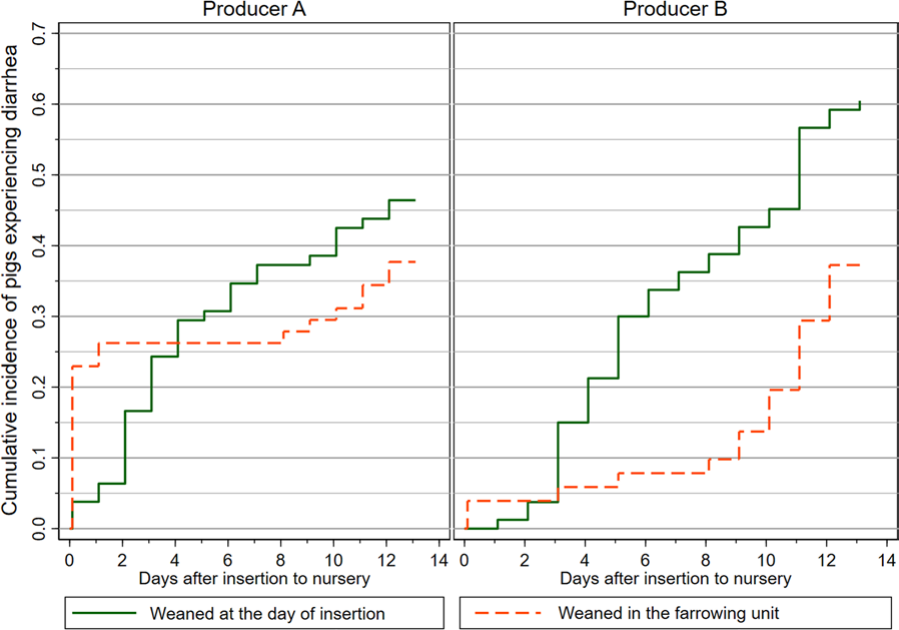
The cumulative incidence of diarrhea divided on time of weaning. The cumulative incidence of diarrhea (Kaplan–Meier failure estimates) as a function of time since insertion in the nursery unit (days) at two Danish indoor productions (A and B). The curves are grouped by the time of weaning, i.e. pigs weaned at the day of insertion (green solid curve) and pigs weaned in the farrowing unit 2–6 days before insertion (dashed orange curve). All pigs (n = 141 and 131) were assumed to be non-diarrheic at the time of insertion (time = 0), and the first clinical exam was performed at time = 0.1

A Cox proportional hazards model was fitted with the time-at-risk starting at the first clinical examination at the day of insertion. Thus, only the pigs which were non-diarrheic at this time point could enter the study (n = 252, failures = 107, pig days at risk = 2594). *Dam* was included as shared frailty effect in the model, and the coefficients from this analysis is presented in Table 6. The covariate *Producer* was omitted as all the between-producer variance was taken up by the *Pen* covariate. Low birth weight (< 1100 g) and being offspring from young sows were revealed to be possible risk factors. Initial model-building revealed that the assumption of proportional hazards was violated for the covariate *time of weaning*, and therefore was made a time-varying effect*;* being *weaned in the farrowing unit* had a protective effect the first 8 days after insertion (HR = 0.11). This tendency is clear from Fig. 7 displaying a plot of the Kaplan–Meier failure estimates stratified on *time of weaning* at each producer. The hazard rate was approximately constant for pigs that were *weaned at the day of insertion* (dashed orange curves, Fig. 7). At time 0.1, the hazard rate was high for pigs *weaned in the farrowing unit*, especially in nursery A. Apart from these prevalent diarrhea cases at the day of insertion (which were not included in the model), the pigs *weaned in the farrowing unit* had a low hazard rate until the eighth day after insertion at both producers (Fig. 7. From day eight and onwards, failures of the early-weaned pigs started to follow the same pattern as pigs weaned at the scheduled day of insertion (Fig. 7). The pseudo-r^2^ for the model was 0.346 (CI: 0.258, 0.523), indicating that the model had a moderate ability to explain the variance in the data.

**Table 6:** Estimates from a Cox proportional hazards model exploring the hazard of developing post-weaning diarrhea

	Hazard ratio	Std. err	*p* value	95% conf. interval	
Birth weight					
Normal–high (1110–2080 g)	1.00	(Base)			
Low (540–1100 g)	2.30	0.57	0.001	1.41	3.74
Time of weaning					
Weaned at day of insertion to nursery	1.00	(Base)			
Weaned in the farrowing unit, 0–7 days post insertion	0.11	0.06	2.3e^−5^	0.04	0.30
Weaned in the farrowing unit, 8–13 days post insertion	0.84	0.26	0.569	0.46	1.53
Dam parity	0.88	0.05	0.041	0.78	0.99
Pen in the nursery (producer, mean body weight at insertion)					
1 (A, 8.1 kg)	1.00	(base)			
2 (A, 6.5 kg)	0.53	0.24	0.153	0.22	1.27
3 (A, 4.2 kg)	0.16	0.08	3.5e^−4^	0.06	0.44
4 (A, 5.5 kg)	0.61	0.23	0.196	0.28	1.29
5 (B, 4.8 kg)	0.82	0.31	0.605	0.39	1.73
6 (B, 6.5 kg)	0.62	0.23	0.199	0.30	1.29
7 (B, 6.5 kg)	0.82	0.32	0.607	0.39	1.74
8 (B, 8.4 kg)	1.18	0.42	0.652	0.58	2.39

Piglets (n = 251) were followed during first 14 days after insertion in two nursery units at commercial Danish indoor producers

### Average daily weight gain

A mixed effects linear regression model explored the factors explaining weight gain. The final model contained seven fixed effects listed in Table 7, and *Sow/pen before* weaning was selected (over *dam*) as the random effect. None of the tested interactions were kept in the model. The model explained just over half (52.0%) of the total variation in average daily weight gain. Producer explained a large part of the variation in the weight gain (35.5 g/day). Pigs were allocated to pens according to their approximate body size at insertion to the nursery, and therefore some of the variation explained by pen in nursery could be related to weight at insertion. Having diarrhea in 2 days or more was associated with an increased growth rate of approximately 26 g/day. The coefficient for weight at birth was 0.052 g/day. This means that the average daily weight gain will increase 5.2 g if the birth weight increases 100 g. If applying this coefficient with all other predictors being equal, the heaviest piglet (birth weight: 2080 g) would have an increase in daily weight gain of 81.1 g/day compared to the lightest piglet surviving to the end of the study (birth weight: 540 g). Having low body condition score at insertion or being weaned in the farrowing unit predicted substantial increases of the growth rate, but the confidence intervals indicated a wide span of uncertainty for these estimates. The clinical sign hollow flanks was frequently observed in both nursery A (n = 494, 26.0% CI 24.0, 28.0) and nursery B (n = 872, 48.9% CI 46.6%, 51.3%).

**Table 7:** Mixed effects linear regression explaining the average daily weight gain (g/day)

	Coefficient (g/day)	*p* value	95% confidence interval	
Ran. effect: Sow/pen at weaning		8.0 e^−4^		
Constant	94.3	1.1 e^−5^	52.3	136.3
Producer				
Producer A	Baseline			
Producer B	35.5	0.006	10.3	60.6
Weight at birth (g)	0.052	7.6 e^−6^	0.029	0.075
Time of weaning				
Weaned at the insertion day	Baseline			
Weaned in the farrowing unit	18.8	0.018	3.2	34.3
Body condition score at insertion				
Normal or above	Baseline			
Below normal	20.6	0.016	3.8	37.4
Days with hollow flanks	− 7.3	1.0 e^−14^	− 9.1	− 5.4
Days suffering from diarrhea				
0 days	Baseline			
1 days	3.7	0.641	− 11.8	19.1
2 days	26.0	0.001	10.7	41.2
3 days	28.4	0.004	9.2	47.6
4–6 days	23.2	0.052	− 0.2	46.7
Pen in the nursery^a^				
1 (A, 8.1 kg)	Baseline			
2 (A, 6.5 kg)	7.7	0.493	− 14.3	29.7
3 (A, 4.2 kg)	0.9	0.951	− 26.8	28.5
4 (A, 5.5 kg)	-20.4	0.073	− 42.6	1.9
5 (B, 4.8 kg)	-16.2	0.196	− 40.7	8.3
6 (B, 6.5 kg)	1.2	0.918	− -21.1	23.5
7 (B, 6.5 kg)	19.7	0.091	− 3.1	42.6
8 (B, 8.4 kg)	0.0			

Average daily weight gain was measured for the first 14 days after insertion to the nursery pigs units at two Danish indoor producers. n = 258

^a^Pen number (producer, mean weight at insertion)

## Discussion

This study estimated the cumulative incidences of diarrhea associated with *E. coli* and rotavirus A during the first 14 days after insertion to the nursery units in two Danish intensive indoor productions weaning without medicinal zinc oxide. Three risk factors for PWD were indicated, and several factors, including days suffering from diarrhea, explained the variance in growth rate. In the following paragraphs, we interpret our findings and discuss the possible implications for prudent use of antimicrobials and PWD prevention strategies. Finally, we discuss some of the limitations of the study.

### Interpretation of findings

#### Assumed etiologies and pathogen dynamics

The present study demonstrated two interesting cases of infection dynamics in PWD outbreaks in pigs where neither antibiotics nor medicinal zinc were used as a preventive measure. The vast majority of diarrhea cases occurring during the first week after insertion to the nursery were associated with rotavirus A. We suggest that the predisposing factors reviewed in the introduction such as change of diet, post-weaning anorexia, and loss of local lactogenic mucosal immunity against rotavirus A have allowed the virus to flourish in these pigs. Being *weaned in the farrowing unit* seemed to be a protective factor (HRR = 0.11) the first week after insertion to the nursery (Table 6), where rotavirus-associated diarrhea dominated (Fig. 6). We hypothesize that pigs being weaned in the farrowing unit have already experienced rotavirus associated diarrhea, and started to develop immunity at the time of insertion. This explanation is supported by the large proportion of pigs with prevalent rotavirus-associated diarrhea at the time of insertion in nursery A. Future epidemiological studies of PWD in productions with frequent use of foster sows (and thus irregular weaning) should therefore examine pigs from the exact time of weaning rather than insertion to the nursery.

Most of the diarrhea cases were not associated with ETEC detection. Especially in nursery B, ETEC was rarely detected, but instead a large proportion of diarrhea cases was associated with substantial excretion of hemolytic *E. coli* where none of the three major toxins in ETEC were detected. This could reflect a mere association confounded by time since weaning, rather than a causal relation, but our Poisson regression (model 3) estimated an increased incidence rate of diarrhea in pigs shedding hemolytic *E. coli* without toxins, and even higher rate in pigs shedding ETEC (Table 3). Results in accordance with this, was found in early experimental study of the role of virulence factors in porcine colibacillosis where *E. coli* strains were modified using plasmids. Oral inoculation with *E. coli* carrying both enterotoxins and fimbria frequently (n = 20/25) produced severe diarrhea in day-old piglets, while strains lacking these virulence factors did not produce disease (n = 0/8). Interestingly, *E. coli* only carrying fimbria, but not enterotoxins, also produced disease but with a lower morbidity (n = 6/20) and severity [38]. A frequent occurrence of PWD with no detection of ETEC has previously been demonstrated in a European survey including samples from 844 pigs. ETEC was not detected in 59.6% of the 280 investigated farms/PWD outbreaks [11]. This increasing evidence of post-weaning without ETEC-detection is contradicting the common perception of the disease etiology. In a series of semi-structured interviews, Danish pig producers and veterinarians commonly referred to PWD as *“coli-diarrhea”* [39].

Intestinal dysbiosis has been defined as, “a marked decrease in the representation of obligate anaerobic bacteria and an increased relative abundance of facultative anaerobic bacteria belonging to the family Enterobacteriaceae” [40]. Thus, the high fecal excretion of hemolytic *E. coli* could possibly be an indicator of an intestinal dysbiosis. Gut inflammation provides a nitrate rich environment, making nitrate-reducing *E. coli* thrive, and by this mechanism gut inflammation has been hypothesized to cause the shift towards Enterobacteriaceae termed dysbiosis [40]. Gresse et al. [4] refined the hypothesis on causation and suggested that weaning-induced dysbiosis and gut inflammation interplays and enhances each other in an escalating vicious circle. Independently of whether their increased abundance is a cause or an effect, hemolytic *E. coli* could still serve as an indicator for dysbiosis. Hence, the diarrhea cases associated with substantial excretion of hemolytic *E. coli* could be caused by a dysbiosis by the pathogenesis suggested by Gresse et al. [4]. As previously suggested [41] this calls for studies integrating recordings of the gut microbiome, metabolome and pathology to disentangle the causal webs of post-weaning gut health and disease. It may also be that the hemolytic non-ETEC *E. coli* carry a toxin, which has so far not been associated with PWD. For example, a high proportion of *E. coli* from PWD in Denmark has been shown to carry the *astA* gene, encoding the enteroaggregative *E. coli* heat-stable enterotoxin (EAST1) [42]. However, the pathogenicity of this toxin is unclear, and the available research indicates that it is not contributing to the development of porcine PWD [43, 44].

Taken together, our microbiological findings are in agreement with three previous studies demonstrating rotavirus shortly after weaning followed by proliferation of hemolytic *E. coli* [14, 45, 46] even though the shedding of rotavirus was delayed and occurred after proliferation of hemolytic *E. coli* in one of the two trials reported by Hampson and Smith [46].

#### Risk factors for post-weaning diarrhea

Our data suggested that piglets with low birth weight and offspring of younger sows have increased risk of PWD. Given the limited sample size and our explorative model building strategy, the study does not provide conclusive evidence. Yet, both findings are biologically meaningful. For instance, it is well-established that low birth weight is predisposing for many negative health outcomes such as pre-weaning mortality [47]. Multiple studies have investigated herd-level risk factors for PWD (e.g. [48, 49]), but to our knowledge, research exploring (early-life) risk factors for PWD at individual pig-level have been sparse prior to our study. A review paper from 2017 dealt with risk factors for *E. coli*-associated PWD, but the statements about factors related to the individual pig (e.g. parity of the dam) were founded in a generalization of evidence about pre-weaning health [50]. Dou et al. [51] had reported differences in the gut microbiota 7 days after birth in 13 pigs that experienced PWD compared to seven pigs that stayed healthy throughout the post-weaning period. Therefore, a specific motivation for the present study was to collect evidence clarifying the hypothesis, that pre-weaning diarrhea increases the risk of experiencing PWD in an individual pig. However, few piglets (n = 7, 2.5%) had pre-weaning diarrhea recorded, and in line with this, the post-mortem exams revealed that gastrointestinal lesions were infrequently assigned as the primary cause of death in the pre-weaning period. Consequently, the data material did not allow us to make inferences regarding the effect of pre-weaning diarrhea on the risk/hazard of developing PWD.

#### Growth rate

A previous study has shown that the pen-level occurrence of PWD was negatively associated to the average weight gain of the piglets within the pens [48]. Our study of individual pigs was contradicting this, at least for pigs having diarrhea for more than just a single day. A reasonable explanation could be that high feed intake (“overeating”) confounds the association, as it both increases the risk of post-weaning diarrhea [46] and improves growth. It should also be noted that it is actually the combined effect of suffering from diarrhea and receiving antimicrobials we have studied. Both of the suggested explanations call for further research.

The observed influence of birth weight on growth rate likely reflects the importance of genetic factors as well as a long-term negative impact of intrauterine growth restriction, which is strongly associated with low birth weight [52]. Piglets with low birth weight, and/or intrauterine growth restriction syndrome are known to have poor growth, impaired ability to resist disease and a higher pre-weaning mortality [52–55]. The piglets weaned before the insertion day had a higher growth rate than piglets weaned at the day of insertion. This might reflect that the farm personnel picked the most robust piglets (with best growing potential) for early weaning. Furthermore, weaning is often accompanied with a period of adaption (to new feed etc.) leading to poor or negative weight gain [56, 57]. The pigs *weaned in the farrowing unit* had already been through these first challenging days when they were inserted to the nursery. This explanation was echoed in a negative average growth rate during the few [1–6] days from weaning to insertion to the nursery in the early-weaned pigs (mean: − 5.3 g/day, SD: 91.0, results not shown). The explanation would also be in line with the finding that *Body condition score below normal at the day of insertion* was associated with an increased growth rate. We hypothesize that pigs with poor body condition score have been diseased or living under suboptimal conditions prior to insertion to the nursery e.g. staying with a sow with poor milk production or have been weaned in the farrowing unit (*weaned in farrowing unit* was associated with *BCS below normal at insertion*: Crude relative risk = 2.4, *p* = 0.005). These pigs might prosper after insertion to the nursery where they will benefit from the liberal feed availability or that they have already lived through the toughest first days after weaning. Evidence of such compensatory growth have been presented previously. Huting et al. [58] showed that pigs with intermediate or high birth weight, but low weaning weight, had higher odds of being heavy at the age of 99 days compared to pigs with low birth weight.

Hollow flanks is, to the authors’ knowledge, commonly interpreted by pig practitioners as a sign of lacking feed intake in weaners and growers. The results indicated that this interpretation is reasonable, as the variable was associated with a decreased average daily weight gain.

### Implications for prudent antimicrobial use and prevention strategies

The present study demonstrated two cases of PWD outbreaks, where the justification for oral batch medication with antimicrobials was questionable. A substantial proportion of the pigs stayed healthy, a large proportion of the cases were associated with a non-bacterial pathogen (rotavirus), and only a minority of the cases were associated with the pathogenic bacterial agent (ETEC) that was the indication for the veterinarians’ prescriptions and the producers’ common initiation of oral antimicrobial batch medications. Antimicrobials affect the gut microbiota composition [59] and by this mechanism, some might advocate that treatment could hypothetically relieve clinical disease associated with dysbiosis and therefore they should still be administered. Even if future research was to support this hypothesis, we argue that prudent use entails promotion of gut symbiosis and health by alternative measures, and reserving antimicrobials for treatment of diseases caused by a bacterial infection. In essence, our study has a sample size of n = 2, when inferring about diarrhea outbreaks as a whole. To which extent the etiologic and disease dynamic patterns, like the two seen in this study, are prevalent across the population of nursery pig units is unknown. Nevertheless, the study demonstrated the existence of PWD outbreaks where ETEC did not play a major role as causative agent.

The study also demonstrated that outbreaks of PWD might have a biphasic pattern. In both productions, we saw two peaks in diarrhea prevalence, where rotavirus dominated at first followed by *E. coli*-associated cases. Another study has demonstrated a high within-herd between-batch variation of the three dominating pathogens (*Lawsonia intracelluaris*, *Brachyspira pilosicoli* and ETEC) in outbreaks of porcine intestinal disease in nursery pigs 10–70 days after weaning [34]. Consequently, the necessity of frequent microbiological laboratory diagnostics to appropriately select antimicrobial substance has been proposed [60]. The two cases of within-outbreak shift in dominating etiology presented in this paper further complicates diagnosing and treating intestinal diseases in flocks of pigs. Practitioners must not only consider between-outbreak variation in pathogens, but also shifts within (what could be perceived as) one outbreak in a given batch.

The risk factors proposed by our Cox model, suggest that taking extra care for offspring of younger sows and with low birthweight could aid the control of PWD. Previous studies have concluded that it is beneficial to sort piglets based on litters rather than body weight. This will reduce aggression and stress, and the resulting gastrointestinal tract impairment in the early post-weaning period (reviewed by [9, 56]). Our findings demonstrated that disease dynamics might be different between piglets weaned in the farrowing unit, and piglets weaned at the day of insertion to the nursery. Therefore, it could be advisable to sort early-weaned litters into designated pens in the nursery. This might increase the chance of a synchronized course of gut development and infections within a pen. This synchronization could both lower the load of circulating pathogens and increase the possibility of making well-timed metaphylactic treatments. As such, this is a proposal worth to investigate in further research or in herd-specific trials conducted as part of the continuous development of the management at a given farm (e.g. as suggested in [61]).

### Limitations of the study

A limitation to the present study is the relatively small sample size. Furthermore, our regression model building was explorative, in the sense that we fitted models that best described the collected data, rather than testing whether our data fitted a pre-specified hypothesized model. Certain features characterized the study population, e.g. injections with antimicrobials during the first day of life, and a large proportion of cross-fostered piglets. While this is representative for Danish indoor production, it might limit the generalizability to other populations.

We included 10 piglets per sow/litter (n = 30). By this approach, piglets from small litters had a higher probability of being included than piglets from large litters. Consequently, piglets from small litters have been overrepresented in our sample in comparison to the target population. We could have avoided this by including a fraction (e.g. half) of the pigs in each litter rather than a fixed number (n = 10) of pigs per litter. However, model 1 failed to suggest litter size as a possible risk factor for PWD. Further criticism might be valid for the methodology for random inclusion of pigs, which was chosen for its practicality. Even though this was not a perfect random probability sampling with enumeration of all pigs, we believe we have included a representative sample of pigs, for instance the male:female ratio and the distribution of birth weights were as expected if by random selection.

Laboratory diagnostics were conducted to determine which *E. coli* strains were shed in the feces. We presume that our approach had a satisfactory sensitivity for detection of ETEC. ETEC strains most frequently associated with PWD carry the fimbria type F4 or F18 and the toxins heat-labile toxin (LT), heat-stable toxin a (STa) and/or heat-stable toxin b (STb) [11, 62]. F4 and F18 positive *E. coli* colonies almost always express hemolysis when cultured on blood agar, and thus genotyping hemolytic colonies is a sensitive method for detecting ETEC shedding [63, 64]. This has been confirmed in fairly recent strains from diarrhea cases in Danish nursery pigs [65].

## Conclusions

The cumulative incidences of PWD the first 14 days after insertion to nursery units not using medicinal zinc were 41.8% (CI 33.6, 50.4) and 51.1% (CI 42.3, 60.0) at producer A and B, respectively. The occurrence of cases associated to ETEC was low, but rotavirus might be an important contributing cause to PWD. We observed a biphasic pattern in the assumed etiology with rotavirus occurring first, and then a shift towards cases associated to ETEC/non-ETEC hemolytic *E. coli.* Being offspring of older sows was a protective factor for the development of PWD (HR = 0.88 [0.78, 0.99] per 1 unit increase in parity of the dam). Low birth weight reduced the post-weaning growth rate (− 5.2 g/day [2.9–7.5] per 100 g decrease in birthweight) and increased the hazard of developing PWD (HR for birthweight below 1100 g: 2.30 [1.41–3.74]). The combined effect of having diarrhea for 2 days or more and receiving antimicrobial treatment was associated with an increase in the average daily weight gain.

## Methods

**Fig. 8:**
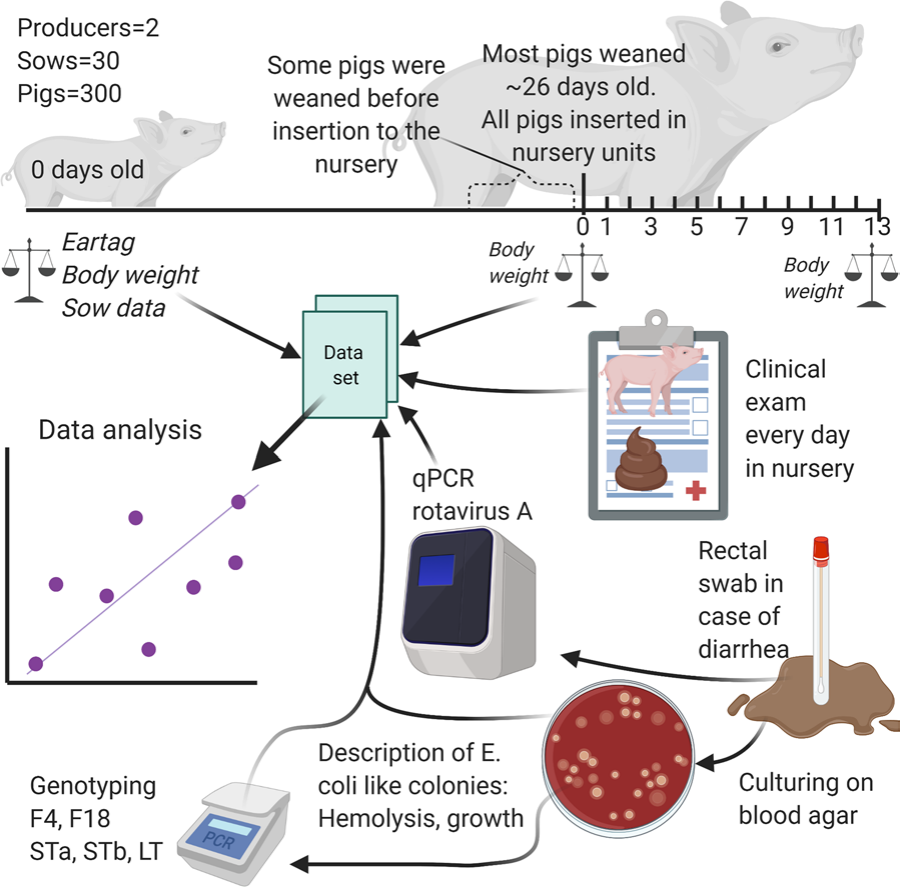
Graphical abstract of the methods in a cohort study of post-weaning diarrhea in pigs. Created with BioRender.com. Pigs were weaned without medicinal zinc and prophylactic antimicrobials. The study investigated risk factors, pathogen dynamics, and association to growth rate

The study was conducted as a cohort study. The paper was drafted to comply with the STROBE-VET guidelines [66]. A summary of the methodology is available in Fig. 8 (created using BioRender.com).

### Study population and setting

A cohort of 300 piglets were followed from birth to 14 days after insertion in the nursery unit. Sample size calculation suggested that 865 pigs were necessary to show an effect of pre-weaning diarrhea on the incidence of PWD, but due to limited resources, only 300 piglets were included. The pigs were kept in two different commercial Danish intensive indoor production systems. Both producers had a multisite structure with a farrowing unit at one location and the nursery unit at another location. The producers were purposely selected among the participants in another study of the causes of PWD [67], as they fulfilled all of the following inclusion criteria: Did not use in-feed medicinal zinc oxide at weaning; experienced problems with post weaning diarrhea associated with fimbria positive *E. coli*; and frequently initiated antimicrobial batch medication against PWD based on clinical signs.

The study period was set to start just before an expected peak in the rate of farrowings, the 7th of September 2019 at producer A, and 21st of September 2019 at producer B. The source population was the first 15 litters to be born in each of the two farrowing units (30 litters in total), and the study population was obtained by randomly selecting ten piglets from each litter. The final fifteenth litter was born on the second day (8th of Sep.) at producer A and the fourth day (24th of Sep.) at producer B. The random selection was performed by collecting all piglets within a given litter in the corner of the pen. Hereafter, 10 piglets were picked up blindly. Cross-fostering and medications were not allowed before inclusion, but some piglets had died or were euthanized during the first hours of life, and these piglets were obviously not given the chance to enter the study.

The majority of the piglets were weaned at the day of insertion to the nursery unit, i.e. at the 26th and 27th day after start of the study at producer A and producer B, respectively. No piglets were weaned later than these days, but many piglets were weaned earlier as their sow was selected to be a foster sow. These early-weaned piglets stayed in the farrowing units, either in a climate controlled section provided for this purpose (producer A) or in their farrowing pens (producer B), until the day of common weaning. At this day, all included pigs were collectively transported to the nursery unit of the respective producer. At each production site, the pigs were allocated to four pens with approximately 30–35 pigs. The pigs were sorted by approximate body size, as it was usual practice in both nursery units, and no pigs were moved during the study period.

### Records and sampling

At the day of birth, the piglets were weighed and their sex recorded. Parity and litter size were noted for the dams. The farm personnel took care of the piglets during the suckling period and registered an indication for treatment before any antimicrobial treatments were initiated. Piglets were weighed at insertion in the nursery and at death or at censoring (i.e. 14 days after insertion to the nursery at the end of the study-period). Fecal consistency [68] and clinical signs were recorded for all pigs every day after insertion in the nursery section. A dichotomous scoring of fecal consistency (diarrhea yes/no) was made based on the appearance of the feces on a rectal swab [69], and if the pig spontaneously defecated, this was taken into account as well. When a new case of diarrhea arose, the given pig was subjected to 3 days of individual oral medication (SID) with neomycin sulfate (Neomay, ScanVet) 25.000 UI/kg body weight/day. If a pig still displayed diarrhea at the end of this treatment, the medication was prolonged for an additional 3 days.

### Necropsy

All piglets that died in the pre-weaning period underwent post-mortem investigation at the Department of Veterinary and Animal Sciences, at the University of Copenhagen. The piglets were stored in a cool room at the farm until collection for necropsy, twice a week. The pigs were subjected to a total necropsy as previously described [70]. All lesions were recorded. All piglets were assigned one primary cause of death based on the lesions recorded, although several pathological manifestations could be present in individual pigs.

### Microbiology

#### Sampling

Whenever a new case of diarrhea was observed, a rectal swab was collected for microbiological analyses. In addition, half of the piglets (even ear tag numbers, n = 75 at each producer, n = 150 in total) were repeatedly sampled for microbiological analyses of their rectal contents irrespective of their fecal consistency. A rectal swab was collected from this sub-cohort routinely every second day (day 0, 2…12) during the first 14 days after the day of insertion into the nursery unit. For practical reasons, this systematical sampling was performed at day − 1 (i.e. the last day in the farrowing unit) instead of day 0 at producer A. All rectal swabs were immediately placed in 1 mL Amies medium (ESwab™, COPAN Diagnostics Inc.). The samples were stored in a refrigerator in the herd, and transported to the laboratory at University of Copenhagen two times weekly. At arrival, a 200 μl subsample was frozen at − 80° Celsius for rotavirus A detection.

#### Bacteriology

At arrival of the sample, 10 μl was plated out on a blood agar (agar base supplemented with 5% calf blood) using an inoculation loop and incubated at 37 °C for 24 h. Subsequently, a visual screening for hemolytic *E. coli*-like colonies was performed. To be considered *E. coli,* colonies had to be greyish-white, medium to large size and mucoid. In any case of doubt, the *E. coli* classification was confirmed by Matrix-Assisted Laser Desorption Ionisation Time-Of-Flight (MALDI-TOF) mass spectrometry [71]. Hemolytic ability, growth extent, and relative abundance compared to other colony types were described for the most dominant hemolytic *E. coli*-like phenotype on the agar plate. A colony of the most dominant *E. coli* phenotype was picked and plated out on another blood agar for purification. If no hemolytic colony was present, the most dominant non-hemolytic *E. coli*-like phenotype was described and purified. Once purified, the cultures were frozen (− 80 °C) in Brain Heart Infusion broth supplemented with 30% glycerol. Hemolytic *E. coli* were further subjected to DNA isolation and multiplex PCR according to a previously described procedure [72] with minor modifications. A total volume of 25 µl PCR reaction mix was prepared as follows: 0.5 µl of each F4(K88), F18, LT, STa and STb forward and reverse primers at a concentration of 100 µM were mixed with 12 µl of DreamTaq Green PCR Master Mix (2x) (Thermofisher Scientific), 6 µl of nuclease-free water and 2 µl of DNA lysate. PCR was completed and products were separated on an electrophoresis gel using conditions as previously described [72].

#### Rotavirus detection

All samples originating from diarrhea cases were analyzed for the presence of rotavirus A. Reverse Transcription real-time PCR (RT-PCR) was carried out as previously described [73]. Nucleic acids were extracted from 200 μl Aimes medium using the Cador Pathogen 96 Qiacube HT kit [5] ref. 54161, automated on the Qiacube HT (Qiagen) according to instructions from the supplier. Three primer and probe sequences [73, 74] targeting rotaviral RNA were diluted in a final volume of 15 μl using an AgPath-ID one-step RT-PCR reagents kit (Applied Biosystems, Foster City, USA) with 3 μL RNA. RT-PCR buffer (2X) (7.5 μL) was mixed with 0.12 μL of each primer (50 μM), 0.18 μL probe (10 μM), 0.6 μL RT-PCR enzyme mix (25X) and nuclease-free water. The thermal cycling conditions were 45 °C for 10 min, 95 °C for 10 min, followed by 48 cycles at 95 °C for 15 s, and finally 60 °C for 45 s. The fluorescence signal was obtained in the green channel during the 60 °C step [73].

### Case-definitions

The result of a bacteriological culturing should be interpret semi-quantitatively when investigating the role of *E. coli* in PWD [64]. We applied the following criteria (both must be fulfilled) to diagnose *abundant of hemolytic E. coli*: Growth of a hemolytic *E. coli*-like phenotype on at least 20% of the plate (i.e. more than *a few colonies*); and the considered hemolytic *E. coli*-like phenotype is making up approximately 50% or more of all colonies on the plate. We defined seven case definitions for the linked presentation of clinical findings and microbiology, and these are presented in Textbox 1. All criteria had to be fulfilled to get a given diagnosis.

**Table:** Textbox 1: Case definitions applied in the cohort study of post-weaning diarrhea

**1. ETEC-associated diarrhea**	
Diarrhea	
Abundant growth of hemolytic *E. coli*	
The considered *E. coli*-like phenotype is confirmed to carry genes for fimbria (F4 and/or F18) and toxins (STa, STb and/or LT)	
**2. Hemolytic non-ETEC** ***E. coli*** **associated diarrhea**	
Diarrhea	
Abundant growth of hemolytic *E. coli*	
The considered *E. coli*-like phenotype is not carrying genes for both fimbria (F4 or F18) and toxins (STa, STb, and/or LT)	
**3. Rotavirus-associated diarrhea:**	
Diarrhea	
Ct-value < 33 for rotavirus A	
**4. ETEC- and Rotavirus-associated diarrhea**	
Fulfills both the case-definition for ETEC-associated diarrhea and Rotavirus-associated diarrhea	
**5. Hemolytic non-ETEC** ***E. coli-*** **and Rotavirus-associated diarrhea**	
Fulfills both the case-definition for Hemolytic *E. coli* associated diarrhea Rotavirus-associated diarrhea	
**6. Diarrhea with no detection of pathogens**	
Diarrhea	
Complete test results for both rotavirus and bacteriology is available	
Does not fulfill the criteria for ETEC-associated diarrhea, hemolytic *E. coli* associated diarrhea, or Rotavirus-associated diarrhea	
**7. Diarrhea with unknown etiology**	
Diarrhea	
Missing test results for either rotavirus or bacteriology, and no reasonable imputations could be made	

### Statistical analyses

#### Descriptive statistics and simple analyses

All statistical work was carried in Stata IC 16 [75]. The distributions of continuous variables was evaluated with histograms and quantile–quantile plots. Descriptive statistics including proportions and means or percentiles (in case of non-normality) were produced for all variables. Measures of disease frequency were estimated for first cases of diarrhea, and the number of second cases were calculated. A pig was considered at risk of experiencing a second case when it had experienced a first case of diarrhea, followed by at least one intermediate day without signs of diarrhea and it was least 24 h since the last neomycin administration. Kaplan–Meier failure estimate plots were created per producer for the following events after insertion to the nursery: First cases of diarrhea, first cases of diarrhea divided by the seven case definitions (i.e. assumed microbiological etiologies), shedding of hemolytic *E. coli*, shedding of ETEC and time of weaning. In these Kaplan–Meier estimates, all pigs were assumed to be healthy at the time of insertion to the nursery (t = 0), and thus prevalent diarrhea cases at the first clinical exam (t = 0.1) were included as a failure (as opposed to left truncating them). The Kaplan–Meier failure function was also used to summarize the time-to-cure divided on producer, and on etiologies if n > 10 (thus only single-etiology cases). A pig was considered at risk of being cured, when it experienced its first case of diarrhea and received a subsequent antimicrobial treatment, and cure was defined as not having diarrhea at a clinical examination. Prevalent cases at the day of insertion was not included in this estimation.

#### Regression models

Three regression models were built (guided by [76]) to serve different scientific objectives:

- *Model 1* To explore whether any covariates were associated with an altered hazard of experiencing an event of PWD.
- *Model 2* To explore whether any factors were associated with an altered growth rate after insertion in the nursery unit.
- *Model 3* To explore effect of shedding *E. coli* with different virulence factors on the incidence rate of diarrhea cases.

##### Model 1: Cox model for exploration of risk factors

To serve the objective of Model 1 we built a Cox proportional hazards model [77]. The time-at-risk was set to start at the time of the first clinical exam, and pigs with prevalent diarrhea at the day of insertion were not included in the analysis. Tied events were handled by the Efron method.

The covariates that were considered for the model, are listed in Table 8. To prevent multicollinearity, all covariates were evaluated pairwise for associations/correlations. *Producer* and *pen* were controlled for by adding them as covariates, and *Dam* was added as a shared frailty*.* The covariate *body condition score at the day of insertion* was considered an indicator of the health of the piglet at the time of insertion. Due to low variance, this predictor was dichotomized into the categories: *normal or above* (89.7% n = 227) and *below normal* (10.3%, n = 26)*.* The clinical signs, *hair coat* and *general condition,* were not considered due to low variability (prevalence at t0: 1.1% n = 3 and 0.4% n = 1, respectively). A plot of martingale residuals indicated a non-linear tendency for birth weight: A birth weight up to approximately 950 g was associated with a constant, increased hazard; from approximately 950 g to 1300 g the hazard decreased; and reached a constant, lowered level when above approximately 1300 g. Previous studies (as meta-analyzed in [47]) have demonstrated a similar trend in the risk of pre-weaning mortality, and a birthweight below 1110 g has been suggested as a threshold for increased risk of pre-weaning mortality [47]. Therefore, the variable was dichotomized into *low birthweight* (540–1100 g), and *normal or high birthweight* (1110–2080 g).

**Table 8:** Variables considered for inclusion in two regression models in the cohort study of post-weaning diarrhea

Variables	Scale of measurement	Model 1 (Cox PH)	Model 2 (Mixed linear)
Producer	Dichotomous (A, B)	Covariate	Fixed effect
Pen in nursery	Nominal (n = 8)	Covariate	Fixed effect
Sow/pen before weaning	Nominal (n = 45)		Random effect
Dam	Nominal (n = 30)	Shared frailty	Random effect
Dam parity	Discrete (1–7)	Covariate	Fixed effect
Total (still + live born) litter size, dam	Discrete (total n pigs 14–28)	Covariate	Fixed effect
Proportion of litter stillborn	Proportion (0–1)	Covariate	Fixed effect
Sex at insertion to nursery	Nominal (castrated male, intact male, female)	Covariate	Fixed effect
Weight at birth	Continuous (grams)	Covariate	Fixed effect
Weight at birth, dichotomized	Dichotomous (low birth weight, normal or high birth weight)	Covariate	
Moved in the farrowing unit	Dichotomous (stayed with dam, moved)	Covariate	Fixed effect
Time of weaning	Dichotomous (weaned in the farrowing unit, weaned at insertion to nursery) A Time varying effect was added	Covariate	Fixed effect
Weight at insertion	Continuous (grams)	Covariate	Fixed effect
Body condition score at insertion to nursery	Dichotomous (normal, below normal)	Covariate	Fixed effect
Days with hollow flanks	Discrete (n days with hollow flanks 0–14)		Fixed effect
Days suffering from diarrhea	Nominal: (0 days, 1 day, 2 days, 3 days, 4–6 days)		Fixed effect

Variables considered for inclusion in two regression models investigating risk factors for porcine post-weaning diarrhea (Model 1, Cox proportional hazards model), and factors associated with altered growth rate the first 14 days after insertion to the nursery unit (Model 2, mixed linear regression model)

A backwards stepwise selection procedure was applied where covariates with *p* < 0.05 were kept in the model. Excluded covariates were added back into the model one at a time to check for confounding effects. Violation of the proportional hazards assumption was checked by testing for a non-zero slope when plotting Schoenfeld residuals against time. Furthermore, parallelism of curves was graphically assessed in ln-cumulative hazard plots for categorical covariates, and by plotting Schoenfeld residuals with a smoothed line for continuous covariates. The assumption of proportional hazards was violated for *weaned in the farrowing unit*, and this was handled by making it a time varying effect. The linearity of relationships with continues and discrete covariates were checked by estimating martingale residuals from the final model without the covariate. Following this, the residuals were plotted against the given covariate with a lowess smoothed curve. Deviance residuals were plotted against analysis time to check for outliers. An r^2^ statistic [78, 79] was calculated to assess the variation explained by the model.

##### Model 2: Linear regression of effects on growth rate

Model 2 was built as a multilevel mixed-effects linear regression. Only pigs that survived until 14 days after insertion and had no missing values for the considered predictors were included (n = 256). The outcome variable was the *average daily weight gain (g)* the first 14 days after insertion to the nursery unit. Biologically relevant covariates were considered for the model (Table 8). *Sow/pen before weaning* and *dam* were highly correlated, yet not organized in a hierarchy. Therefore, the variable that explained most of the variance in a univariable linear regression was added as a random factor and the other was excluded from the analysis. Both *producer* and *pen in the nursery unit* were controlled for by adding them as fixed effects. Two variables, *hollow flanks days* and *diarrhea days,* were created to reflect the time of the post-weaning period with low feed-intake and time suffering from diarrhea, respectively. *Hollow flanks days* was created by dichotomizing the observation of abdominal shape into hollow flanks/not hollow flanks (including normal and distended abdomen), as there were only two observations of abdominal distention. This variable was treated as continuous, i.e. number of days suffering from hollow flanks (0–14), as a lowess smoothed curve indicated a linearity of the relationship to average daily gain. Likewise, *diarrhea days* summarized the number of days a given pig had suffered from diarrhea (ranging from 0 to 6). However, this variable was made categorical to allow the combination of 4, 5, and 6 days, as these occurred relatively infrequently (n = 9, 3, and 3, respectively). A backwards selection procedure, and a check for confounders and interactions were performed as done for Model 1. The final model was reestimated without *birth weight* and *time of weaning* to assess if these variables were colliders between *diarrhea days* and *weight gain*. The assumptions for the model were evaluated at both pig-level and at the level of the random factor (*sow/pen before weaning*)*.* The assumption of normality of the residuals was evaluated graphically (histogram and normal quantile plot) and by Shapiro–Wilk test for normality. The homoscedasticity of the error was evaluated by plotting standardized residuals against the predicted average daily gain.

##### Model 3: Poisson regression exploring the importance of E. coli virulence factors

Based on the pigs that had been systematically sampled every second day, a four-level nominal variable was created by grouping the hemolytic *E. coli* strains by the presence (±) of fimbria, and toxins. The incidence rate ratios between the four groups were estimated with a Poisson regression model. The model was based on observations of the systematically sampled half of the cohort. The outcome variable was the count of new diarrhea cases, and both first and second cases of diarrhea were included. Producer was added as a covariate. Adding pen as random effect did not affect the model, and therefore this factor was omitted. The exposure (risk time) was measured in pig days, and a pig was not considered at risk when it had received neomycin within 24 h. Each bacteriological culturing contributed with up to two pig days, the sampling day and the following day (unless a new sample was cultured that day). The first 6 days after insertion to the nursery, the pigs generally (622 out of 692 pig days) shed non-hemolytic *E. coli*, and strains with fimbria and/or toxins were rare (12 pig days). Therefore, time-since-insertion (and rotavirus involvement in diarrhea cases) confounded the variable describing types of *E. coli*. Correspondingly, plotting of the ln-survivor probability against analysis time revealed that the failure rate for pigs shedding non-hemolytic *E. coli* was high at first, but rapidly decreased and became constant(= 0) after the seventh day after insertion. For these reasons, it was most appropriate to restrictively include observations from the seventh day after the insertion and onwards. Goodness-of-fit tests of the model was performed based on both Pearson and deviance residuals.

## Declarations and transparency

Some of the data included in this publication have been presented in preliminary forms in three master theses submitted to University of Copenhagen in 2020. Oleksandr Kolesnyk and co-author of this paper KTH contributed to the post-mortem exams and some findings were described in their cooperative master thesis. The co-authors MVA and AEC described clinical and microbiological findings in preliminary forms in their master theses. Some of the samples collected during the present work will be analyzed for other purposes presented in separate papers. A Danish resume with preliminary findings have been published previously [80].

### Ethics statement

The principal investigators deemed that the procedures performed would not cause pain, suffering, distress, or lasting harm to an extent where an approval for experimental animal use was necessary according to Danish law [81]. Producer B’s own records revealed that some pigs had been weaned when 20 days old. This is not legal practice in Denmark unless due to health or welfare issues (28 days are required, but 21 days of age is permitted when specific requirements to the production system is fulfilled) [82]. In accordance with §8 in the Danish veterinary act [83], the veterinarian responsible for the data collection instructed the producer to take actions that would preclude this practice in the future.

## Supplementary Information

**Additional file 1**. Additional tables with summary of dams and their litters, and primary causes of pre-weaning death.

## Data Availability

The data set is available from the first author upon reasonable request.
