# Supplementary material for: Post-weaning diarrhea in pigs weaned without medicinal zinc: risk factors, pathogen dynamics, and association to growth rate
Source: Porcine Health Manag. 2021 Oct 9;7:54. doi: 10.1186/s40813-021-00232-z (PMC8501929; doi:10.1186/s40813-021-00232-z)
Supplement: Supplementary file 1 — Additional file 1. Additional tables with summary of dams and their litters, and primary causes of pre-weaning death. [file 40813_2021_232_MOESM1_ESM.docx]

# Supplementary material

Table S1: Characteristics of litters and dams (n=30) included in a cohort study of post-weaning diarrhea in two commercial Danish indoor pig productions

|  | **Producer A** | **Producer B** |
| --- | --- | --- |
| Mean % live born piglets | 90.4 % | 89.6 % |
| **Litter size (total*)** |  |  |
| Mean | 22.6 | 21.1 |
| Minimum | 18 | 14 |
| 25% percentile | 20 | 17 |
| 50% percentile | 23 | 21 |
| 75% percentile | 25 | 25 |
| Maximum | 28 | 26 |
| **Sow Parity** |  |  |
| 1 | 3 (20%) | 5 (12.5%) |
| 2 | 3 (20%) | 5 (12.5%) |
| 3 | 4 (26.7%) | 6 (12.5%) |
| 4 | 0 | 2 (12.5%) |
| 5 | 4 (26.7%) | 6 (12.5%) |
| 6 | 1 (6.7%) | 5 (26.7%) |
| 7 | 0 | 1 (6.7%) |

*total= n stillbirths + n live born

Table S2: Number (%) of piglets and the primary causes of death in the pre-weaning period according to macroscopic post-mortem evaluations

| **Cause of death** | **Pathological findings** | **Prod. A** | **Prod. B** | **Total** |
| --- | --- | --- | --- | --- |
| **Crushed by sow** | Generalised sub-cutaneus edema, internal lacerations, bone fracture, hemoabdomen, hemothorax, haemorrhagic musculature and subcutis. | 2 (22.2) | 8 (44.4) | 10 (37.0) |
| **Starvation** | Lean, air-filled ventricle, sparse or absent content in the gastrointestinal tract, and crystal-formation in kidneys +/- bladder. | 0 | 4 (22.2) | 4 (14.8) |
| **Other trauma** | Ulceration with exposed musculature on the hind limb. | 0 | 1 (5.6) | 1 (3.7) |
| **Non-infectious causes, total:** | | **2 (22.2)** | **13 (72.2)** | **15 (55.5)** |
| **Polyserositis** | ≥ 2 of the following findings:  fibrinous peritonitis, fibrinous pleuritis, fibrinous pericarditis, proliferative polyarthritis. | 0 | 1 (5.6) | 1 (3.7) |
| **Enteritis/ enterocolitis** | Hyperaemic or haemorrhagic intestinal mucosa, +/- abnormal intestinal content. | 1 (11.1) | 1 (5.6) | 2 (7.4) |
| **Polyarthritis** | Inflammatory changes in multiple joints including: purulent exudate in the joint cavity, hyperplasia or hyperaemia of the synovial membrane +/- fibrous periarthritis. | 1 (11.1) | 1 (5.6) | 2 (7.4) |
| **Osteomyelitis** | Purulent inflammation in the medulla of femur and humerus. | 1 (11.1) | 0 | 1 (3.7) |
| **Infectious conditions, total:** | | **3 (33.3)** | **3 (16.7)** | **6 (22.2)** |
| **Unknown** | Abscense of pathological changes or marked autolytic changes making an accurate evaluation impossible. | 4 (44.4) | 2 (11.1) | 6 (22.2) |
